# The Afterlife of Interspecific Indirect Genetic Effects: Genotype Interactions Alter Litter Quality with Consequences for Decomposition and Nutrient Dynamics

**DOI:** 10.1371/journal.pone.0053718

**Published:** 2013-01-17

**Authors:** Mark A. Genung, Joseph K. Bailey, Jennifer A. Schweitzer

**Affiliations:** Department of Ecology and Evolutionary Biology, University of Tennessee – Knoxville, Knoxville, Tennessee, United States of America; Estacion Experimental de Zonas Áridas (CSIC), Spain

## Abstract

Aboveground-belowground linkages are recognized as divers of community dynamics and ecosystem processes, but the impacts of plant-neighbor interactions on these linkages are virtually unknown. Plant-neighbor interactions are a type of interspecific indirect genetic effect (IIGE) if the focal plant’s phenotype is altered by the expression of genes in a neighboring heterospecific plant, and IIGEs could persist after plant senescence to affect ecosystem processes. This perspective can provide insight into how plant-neighbor interactions affect evolution, as IIGEs are capable of altering species interactions and community composition over time. Utilizing genotypes of *Solidago altissima* and *Solidago gigantea*, we experimentally tested whether IIGEs that had affected living focal plants would affect litter decomposition rate, as well as nitrogen (N) and phosphorous (P) dynamics after the focal plant senesced. We found that species interactions affected N release and genotype interactions affected P immobilization. From a previous study we knew that neighbor genotype influenced patterns of biomass allocation for focal plants. Here we extend those previous results to show that these changes in biomass allocation altered litter quality, that then altered rates of decomposition and nutrient cycling. Our results provide insights into above- and belowground linkages by showing that, through their effects on plant litter quality (e.g., litter lignin:N), IIGEs can have afterlife effects, tying plant-neighbor interactions to ecosystem processes. This holistic approach advances our understanding of decomposition and nutrient cycling by showing that evolutionary processes (i.e., IIGEs) can influence ecosystem functioning after plant senescence. Because plant traits are determined by the combined effects of genetic and environmental influences, and because these traits are known to affect decomposition and nutrient cycling, we suggest that ecosystem processes can be described as gene-less products of genetic interactions among the species comprising ecological communities.

## Introduction

Until recently, above- and belowground subsystems had been studied separately, but the processes that occur in each subsystem are tightly linked [1]–[3] with plants serving as a major intermediary. Environmental impacts on a plant’s phenotype during the growing season have the potential to cross the “living-dead” barrier when, after senescence, plants shed leaves containing important nutrients that enter the belowground system. These “afterlife” effects describe how species- or genotype-based differences in litter quality (e.g., [4], [5]), interactions with herbivores [6]–[9], ozone [7] and UV radiation [10] will feed back to affect ecosystems [11]. For example, species in habitats with low nutrient availability generally use limited resources efficiently and experience low herbivory, but grow and decompose slowly; this leads to slower rates of nutrient cycling, creating a feedback that further favors plants capable of surviving in nutrient-limited environments [11]. In addition to species differences and herbivore-mediated changes to leaf chemistry, litter quality may also be affected by indirect genetic effects (IGEs), which are modifications to the phenotype of one individual due to the expression of genes in another individual [12]. Although the definition of IGEs [12] restricts the term to intraspecific interactions, the IGEs we refer to here occur between members of different species. These interspecific indirect genetic effects (IIGEs) differ from standard IGEs in the sense that they influence species interactions and community change rather than social evolution [13]. Recent work has also suggested that IIGEs may be a key to linking understanding the selective pressures exerted when organisms interact with, and alter, their biotic environments [14]. IIGEs occur when interactions between plants and their neighbors have a genetic basis, though to our knowledge the possibility that IIGEs could initiate “afterlife” effects remains untested.

Decomposition and nutrient dynamics provide an effective way to test how IIGEs affect ecosystem processes because litter from neighboring plants frequently decomposes together, causing unique outcomes that may synergistically enhance or slow litter decay or nutrient release. This observation is responsible for an extensive literature on the effects of litter mixing (see reviews, [15]–[17]), which presupposes that neighbors interact and examines how decomposition is affected when species with different litter quality (i.e., lignin:N, C:N) decompose together. Many litter mixing studies describe the effects of mixtures as either additive or non-additive, depending upon whether decomposition dynamics in litter mixtures can be predicted using single-species or single-genotype dynamics [9], [14], [17]. “Non-additive” effects result when mixture components interact, either directly through physical and chemical changes to the environment [18], [19], or indirectly by altering decomposer communities [8], [18]. The unpredictable effects of litter mixing on ecosystem processes are common, as 67% and 76% of published studies report non-additive changes to decomposition rate and nutrient release rates, respectively, when species of different litter qualities decompose together [14].

The field of community and ecosystem genetics has shown that, in addition to species variation, genotypic variation can have major impacts above the population level (e.g., [5], [9], [13],[20]–[29]). For example, genotypic variation can cause differences in decomposition as genotypes can produce tissues that vary in leaf toughness, nutrient concentration, lignin concentration, or susceptibility to leaf-modifying arthropods [9], [18], [26], [28]. When different genotypes decompose together, studies have demonstrated significant differences in decomposition and nutrient release rates compared with monocultures (i.e., single genotype treatments), although the effects are often weaker than studies comparing species mixtures [9], [18], [26]. It is important to consider, however, that the chemical properties of leaf litter may be impacted by plant-neighbor interactions during the growing season. Therefore, collecting litter from individual genotypes (or species) that were not grown together and mixing this litter to create experimental treatments (as most previous studies have done) may not provide an accurate picture of how genotype mixtures decompose in natural systems because it does not consider the “afterlife” effects of pre-senescence plant-neighbor interactions. This perspective recognizes the potential role that IIGEs could have on ecosystem processes; this is particularly important given that IIGEs can drive the evolution of the biotic environment [12], [14], meaning that genetic changes in one species could affect the ecosystem processes associated with other species. Specifically, IIGEs would be indicated by significant effects of neighbor genotype identity on focal plant traits during the growing season. Many plant traits can be affected by IIGEs, including aboveground productivity [24],[29]–[31], fitness [31] and belowground productivity [29]. IIGEs can also have “afterlife” effects on ecosystem processes if the focal plant trait in question is linked with an ecosystem response such as decomposition or nutrient cycling. For example, a neighboring plant could alter a focal plant’s rate of nutrient uptake or pattern of biomass allocation [29] that could alter litter inputs from the focal plant. The interpretation of IIGEs in community genetics has changed the way genes are functionally annotated [32], meaning that more information about associated community and ecosystem processes is being attached to particular focal plant genotypes. If pre-senescence plant-neighbor interactions affect plant litter quality (e.g., litter lignin:N), which then alters decomposition and nutrient dynamics, this would indicate that ecosystem processes (i.e., fluxes of energy and nutrients) are gene-less products of the “afterlife” effects of IIGEs.

*Solidago altissima* and *Solidago gigantea* provide a model system for examining the “afterlife” effects of inter-specific genotype litter mixing because 1) genotypic variation in these species has been shown to affect a wide range of community and ecosystem processes [21], [22], [27], [29], [33], 2) *S. altissima* and *S. gigantea* are among the most commonly co-occurring species pairs in the genus *Solidago*, and 3) genotypes of both species display high phenotypic variation. Previous work on interspecific genotype interactions with these individuals of *Solidago* found that neighbor genotype identity affected focal plant rhizome, coarse root, and aboveground biomass, showing strong interspecific interactions among neighbors [29]. For the experiment presented here, we collected leaf litter from these same individuals, growing in the same common garden, to examine whether the growing season effects of neighbor genotype extended to affect ecosystem process. By affecting plant biomass and resource allocation, these interactions may lead to differences in plant chemistry that drive patterns of nutrient dynamics after plant senescence.

In April 2008, a common garden experiment was established at Freels Bend on the reservation of Oak Ridge National Laboratory (Oak Ridge, TN) to examine the community and ecosystem level impacts of IIGEs in a *Solidago* sp. system. This common garden included three locally collected genotypes each of *Solidago altissima* and *Solidago gigantea*. We included genotype monocultures and all possible interspecific combinations of *S. altissima* and *S. gigantea* genotypes. We collected leaf litter from senescing *S. altissima* and *S. gigantea* plants and conducted a decomposition experiment using a “bag-within-a-bag” design that allowed us to independently track the decomposition and nutrient dynamics of different genotypes. We decomposed plants in monoculture (two smaller bags with the same genotype of leaf litter within a larger bag) and in genotype mixtures (one smaller bag with a genotype of *S. altissima* and another smaller bag with a genotype of *S. gigantea*). We placed the bags in an old field neighboring the common garden and collected one third of the bags after two, four, and eight months, and analyzed the bags for percent mass lost, nitrogen dynamics, and phosphorous dynamics (see Methods sections for more details). The overarching question asked in this study is whether IIGEs have “afterlife” effects on ecosystem processes, linking IIGEs and ecosystem ecology. We hypothesized that 1) species level differences in litter quality will lead to species interactions that affect decomposition and nutrient release, and 2) interactions among decomposing genotypes within mixture treatments will cause non-additive patterns of mass loss and nutrient dynamics, due to variation among genotypes in phenotypic traits and the response of decomposers to these traits. Given that neighbor genotype affected focal plant biomass in a previous study [29] that used the same individuals and common garden as the experiment presented here, we also predicted that 3) decomposition and nutrient dynamics of interacting neighbors will be affected by “afterlife” effects, (i.e., the outcome of IIGEs that occurred during the growing season). We found that IIGEs during the growing season changed plant biomass and initial litter quality; these changes had “afterlife” effects on decomposition rate and N immobilization.

## Results

### Initial Litter Chemistry

**Figure 1:**
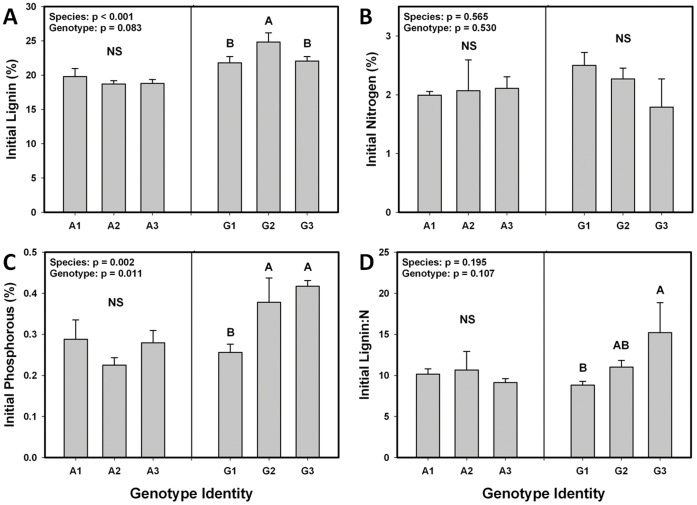
Intraspecific variation affects initial litter chemistry. Initial lignin (A), foliar nitrogen (N) (B), foliar phosphorous (P) (C), and lignin:N (D) values are presented for three genotypes each of *Solidago altissima* and *Solidago gigantea*. “Genotype” p-values refer to genotype nested within species. In addition to this, post-hoc tests were conducted within each species, corrected for multiple comparisons using reverse Bonferroni corrections (α = 0.05), and differences among genotypes within a species are designated by different letters.

We found that initial litter chemistry varied between *S. altissima* and *S. gigantea*, and also among genotypes within *S. gigantea* (**Figure 1**). Initial lignin (Fig. 1a) and P (Fig. 1c) were 21% and 32% higher, respectively, in *S. gigantea* than *S. altissima*. Initial lignin (Fig. 1a), P (Fig. 1c), and lignin:N (Fig. 1d) also differed across *S. gigantea* genotypes, although we observed no association between P and lignin levels. Because we detected species- and genotype-level variation for chemical traits, which are important to decomposition and nutrient dynamics, we would expect to also find species- and genotype-level effects on mass loss and nutrient immobilization and release. Also, because some species and genotypes are of higher nutrient quality (e.g., higher P, lower lignin:N), some litter types may “prime” other litter types.

### Species and Genotype Effects on Decomposition and Nutrient Dynamics

**Figure 2:**
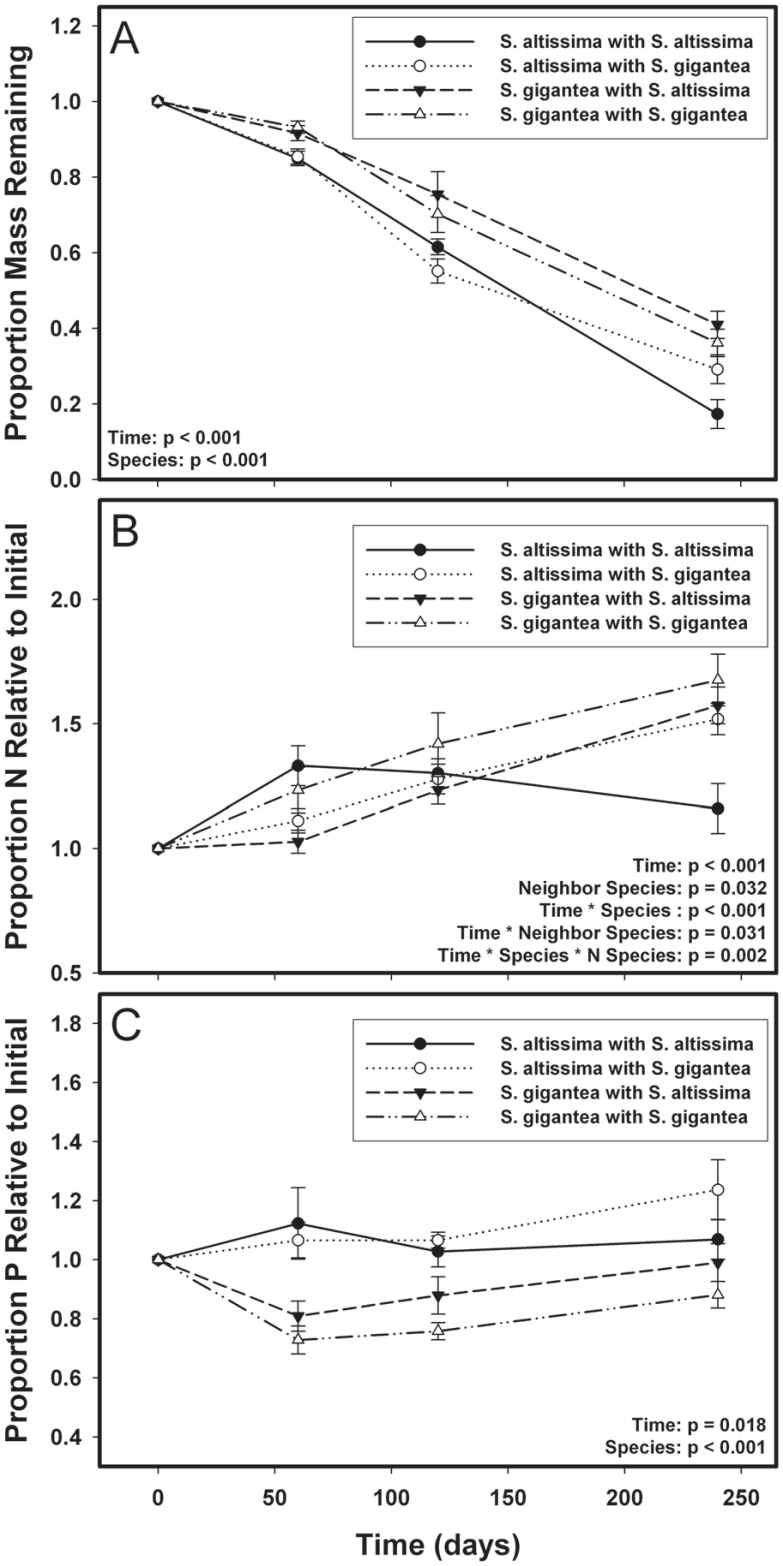
Species-level effects on decomposition and nutrient cycling. At the species level, plant-neighbor interactions drove patterns of decomposition and nutrient release. *Solidago altissima* decomposed faster overall than *Solidago gigantea* (A), a three way interaction between time, species and neighbor species affected nitrogen (N) dynamics (B), and *S. altissima* immobilizes more phosphorous (P) than *S. gigantea* (C). P-values are shown for significant factors (α = 0.05) in a fully factorial design that included time, species identity, and neighbor species identity. Non-significant factors are not listed.

As expected based on initial litter quality, we found that (averaging across all treatments) *S. altissima* decomposed up to 40% faster than *S. gigantea*, although the identity of the neighbor species (for all analyses here, this means the species with which a focal species was decomposed) did not affect mass loss (**Figure 2a** **,** **Table 1**). Species identity also affected P dynamics, as more P was immobilized in *S. altissima* litter than in *S. gigantea* litter (Fig. 2c, Table 1). A three way interaction between focal species, neighbor species, and time (Fig. 2b, Table 1) affected N dynamics. All mixtures immobilized N throughout the experiment; however, in contrast with the other mixtures, rates of N immobilization peaked for *S.altissima* monocultures at two months. Averaged across all collection dates, *S. gigantea* monocultures had N concentrations (relative to initial) approximately 15% higher than the three other treatments (Fig. 2b). In a model containing time, species identity, and genotype nested within species, we found that focal genotype predicted N (p<0.001) and P (p<0.001) dynamics, but not decomposition rate, and did not interact with the “time” factor. These results show that the carbon, N, and P dynamics in *Solidago* spp. were driven, in part, by the identity of both the focal species and the neighbor with which it decomposed, and that N and P dynamics were also affected by focal plant genotype.

**Table 1:** Species effects on decomposition and nutrient dynamics.

Factor	d.f.	Mass Rem. (%) N = 175	N Dynamics (%N/Initial %N) N = 181	P Dynamics (%P/Initial %P) N = 181
		p	p	p
Time	1	***<0.001***	***<0.001***	***0.018***
Species	1	***<0.001***	0.121	***<0.001***
Neighbor Species (NS)	1	0.441	***0.032***	0.533
Time * Species	1	0.118	***<0.001***	0.207
Time * NS	1	0.679	***0.031***	0.262
Species * NS	1	0.550	0.098	0.065
Time * Species * NS	1	0.092	***0.002***	0.167

Results of general linear models relating decomposition and nutrient dynamics to species interactions are shown. Mass remaining and phosphorous (P) dynamics were only affected by time and species. Nitrogen (N) dynamics was affected by a three way interaction of time, species, and neighbor species. Italicized, bolded values are significant at α = 0.05.

### Non-additivity in Genotype Mixtures

**Figure 3:**
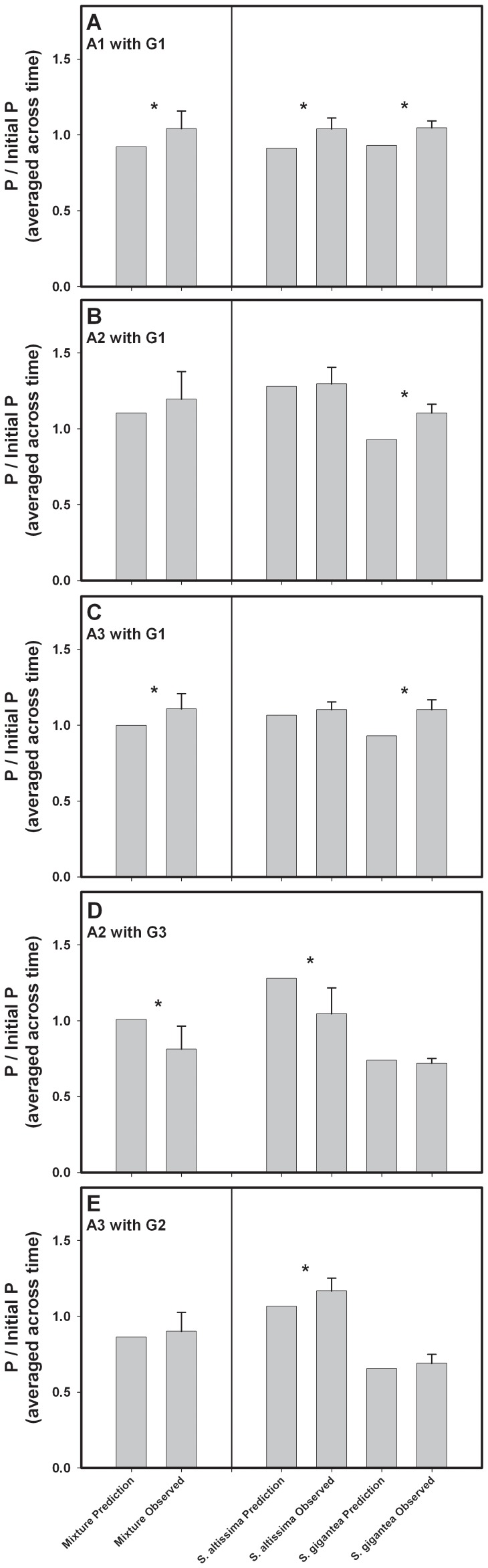
Non-additive effects on phosphorous immobilization. Phosphorous (P) immobilization (averaged across collections) was non-additive for three of five interspecific genotype mixtures. Results are presented at both the mixture level (left panels) and individual genotype level (right panels). For mixtures, asterisks indicate that P immobilization for the mixture as a whole was significantly different from additive expectations based on the monoculture P immobilization of both of the constituent genotypes. For individual genotypes, asterisks indicate that the P immobilization of a focal genotype was different in the presence of a particular interspecific neighbor than in monoculture.

We did not detect non-additive responses for decomposition rate or N dynamics in any of the five interspecific genotype mixtures, suggesting that, for these responses, there were no “priming” effects in litter mixtures. In contrast, three of five genotype mixtures displayed non-additive responses for total P immobilization (**Figure 3**). One of these responses was 19% lower than expected (Fig. 3d), while the other two were 13% and 11% higher than expected (Fig. 3a,c) which is evidence for “priming”. The effect size of interspecific mixtures may be small compared with environmental factors such as temperature and moisture, but understanding the role of genetic interactions on decomposition nonetheless provides additional information about how nutrients are cycled in natural systems. These results may be related to initial litter chemistry, as we detected significant genotype- and species-level effects on initial P (Fig. 1c) but not initial N (Fig. 1b). We did not detect effects of neighbor species or species interactions on P dynamics (Fig. 2c), suggesting that interspecific genotype interactions are relatively more important drivers of P uptake than are species interactions. It is possible that the lack of a species interaction is due to the genotype of one species increasing P immobilization while the genotype of a second species decreases it, making the effects of “species” and “neighbor species” variable and therefore hard to detect. However, this highlights the importance of within-species variation by showing that considering only species-level effects can miss the larger ecological picture.

### “Afterlife” of Interspecific Indirect Genetic Effects

**Figure 4:**
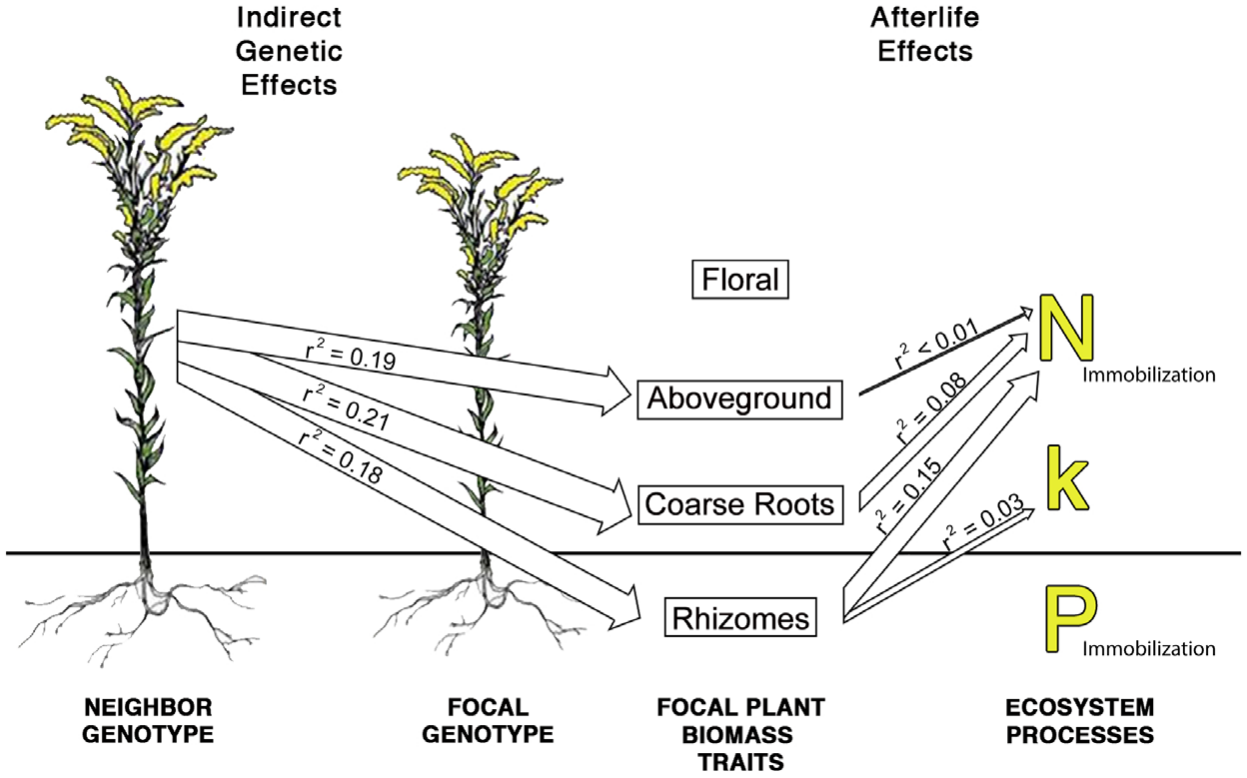
Indirect genetic effects and afterlife effects on ecosystem processes. Interspecific indirect genetic effects (IIGEs) alter focal plant biomass traits, and afterlife effects cross the “living-dead” barrier to influence ecosystem processes. IIGE data is modified from [30], and are not the result of analyses done here. IIGEs show the partial r^2^ values of neighbor genotype on focal plant biomass traits. Ecosystem processes abbreviations represent nitrogen (N) uptake and release, decomposition rate (k), and phosphorous (P) uptake and release. “Afterlife effects” arrows show the partial r^2^ value of plant biomass traits together in a single model that also contained species identity, and genotype nested within species. Arrows are only present for significant relationships. Combined, afterlife effects explained 24% of the variation in N uptake and release.

The results listed thus far only consider the effects of species- and genotype-level variation on ecosystem processes, but we also found several significant relationships through which plant biomass traits (measured in [29] and re-used in the afterlife effects analysis here) crossed the “living-dead” barrier and affected decomposition and nutrient dynamics. Specifically, plant biomass traits affected litter quality, with consequences for decomposition and N and P dynamics. Low rhizome biomass was associated with faster decomposition rates (**Table 2**). Low coarse root biomass, high rhizome biomass, and high aboveground biomass were associated with more N immobilization (Table 2), and these biomass factors explained a total of 24% of the variation in N immobilization even after considering species identity, and genotype nested within species identity. For these afterlife effects, the mechanism is likely changes to plant litter quality due to IIGEs experienced by a focal genotype during the growing season. For example, low coarse root biomass and high aboveground biomass were correlated with lower lignin:N, an indication of higher litter quality (coarse root biomass: LR Χ^2^_(1,16)_ = 4.660, p = 0.031; aboveground biomass; LR Χ^2^_(1,16)_ = 5.129, p = 0.024). However, we did not find any relationship between rhizome biomass and lignin:N (LR Χ^2^_(1,16)_ = 0.761, p = 0.383). Because all of the genotypes used in the decomposition experiment were both grown and decomposed with the same neighbor genotype, and because neighbor genotype is known to affect all of the “biomass” traits listed [29], the above relationships show the “afterlife” effects of pre-senescence IIGEs. For example, *S. altissima* genotype A1 grown in monoculture produced more coarse roots than when it was grown with *S. gigantea* genotype G1 [29]. This led to differences in litter quality that affected decomposition and N immobilization after plant senescence, showing that IIGEs can initiate ecological relationships that influence ecosystem processes (**Figure 4**).

**Table 2:** Contemporary and afterlife effects on ecosystem processes.

Factor		k-constant		N dynamics (%N/Initial %N)		P Dynamics (%P/Initial %P)		Litter Quality (Lignin:N)	
	d.f.	Slope	p	Slope	p	Slope	p	Slope	p
**Contemporary**									
Species	1	NA	***0.038***	NA	0.547	NA	***0.003***	NA	0.680
Genotype [Species]	4	NA	***0.010***	NA	***<0.001***	NA	***<0.001***	NA	0.057
**Afterlife**									
Coarse Root Biomass (g)	1	0.001	0.066	***−0.001***	***<0.001***	0.000	0.804	***0.037***	***0.031***
Rhizomes Biomass (g)	1	***−0.004***	***0.019***	***0.001***	***0.005***	0.000	0.670	0.001	0.383
Aboveground Biomass (g)	1	0.001	0.837	***0.038***	***<0.001***	0.000	0.660	***−0.004***	***0.024***

NA – not applicable.

Results of generalized linear models relating growing season biomass to decomposition rate (k) and nitrogen (N) and phosphorus (P) dynamics are shown. Contemporary effects are factors directly tied to leaf litter decomposition, and afterlife effects are pre-senescence plant traits that may indirect affect decomposition. Neighbor genotype identity is known to have a significant impact on all listed “biomass” factors. All data points are means (N = 16) of a genotype-neighbor genotype pairs (e.g., mean of genotype A1 grown with genotype G2). The slope is the parameter estimate that relates the factors to the ecosystem-level responses, and indicates a positive or negative relationship between the factor and response. Italicized, bolded values are significant at α = 0.05.

## Discussion

Overall, our results indicate that rates of decomposition and subsequent nutrient release are, in part, a legacy of indirect genetic effects (IIGEs) that affected plant phenotypes during the growing season. We found that initial litter chemistry varied between *S. altissima* and *S. gigantea*, and also among genotypes within *S. gigantea* (Fig. 1), leading to *S. altissima* decomposing up to 40% faster than *S. gigantea* (Fig. 2a). Nitrogen dynamics were affected by a three way interaction between species, neighbor species, and time (Fig. 2b), but we did not detect a similar interaction for P dynamics (Fig. 2c). However, we detected non-additive effects of genotype mixing on P dynamics in three of the five genotype mixtures (Fig. 3). In one of the three mixtures, P immobilization was decreased, and in the other two more P was immobilized than expected. We also detected “afterlife” effects that linked the above- and belowground systems, as traits expressed by plants during the growing season were correlated with initial litter quality, decomposition and nutrient dynamics (Table 2, Fig. 4). These traits allow us to describe ecosystem processes as the result of changes in plant biomass driven by IIGEs that occurred before plant senescence.

### Species and Genotype Interactions Influence the Dynamics of Different Nutrients

Species and genotype interactions are ubiquitous in nature and can influence community structure and ecosystem processes, such as decomposition and nutrient dynamics. Our study is the first, to our knowledge, to separately examine the components of interspecific genotype mixtures to determine how decomposition and nutrient dynamics are affected by species and genotype interactions. Our results show that species interactions drive patterns of N immobilization, as we detected a three-way interaction between time, species, and neighbor species. This effect appears to be driven by *S. altissima* monocultures, in which N immobilization rates peaked at two months and then declined (Fig. 2b). The shape of the points describing N dynamics in *S. altissima* monoculture shows that rates of N immobilization decreased over time, although N immobilization was still occurring at the final collection date. This effect may be due to the presence of only high quality *S. altissima* litter; in each of the other three treatments *S. gigantea* litter was present as the focal litter (i.e., the litter in which nutrients were measured), a neighbor litter, or both. Decomposers may have been more attracted to, and able to access N more quickly, in the higher quality *S. altissima* litter leading to an earlier peak in rates of N immobilization. In contrast with the species-level effects, we detected no effects of interspecific genotype interactions on N dynamics. Previous work has suggested that slowly decomposing litter may decompose faster when mixed with higher quality species, due to a higher N flux and more N availability (e.g., [34]). However, we did not detect an increase in *S. gigantea*’s (the lower quality litter) decomposition rate when mixed with *S. altissima* (the higher quality litter). It is possible that we didn’t observe priming effects because the magnitude of the difference in lignin:N between *S. altissima* and *S. gigantea* was small (∼20%) relative to the difference between high and low quality species in other studies of “priming”, which can be over three times that large (e.g., [34]). The smaller difference between *S. altissima* and *S. gigantea* may have been insufficient to elicit a strong response from the decomposer communities.

While at a broad scale it appears that species interactions affect N dynamics, we did not detect an effect of species interactions on P dynamics. Species identity did affect P dynamics, as P was immobilized on *S. altissima* and released from *S. gigantea*; this suggests that decomposer communities were more limited by P on *S. altissima* litter than on *S. gigantea* litter, at least during the early stages of the experiment (Fig. 2c). However, we frequently observed non-additive outcomes for P dynamics in the interspecific genotype mixtures. The non-additive responses were not universal, however, as only certain combinations of genotypes displayed non-additive responses. For example, *S. gigantea* genotype G1 immobilized more P than expected under an additive model in all three mixture treatments in which it was included (Fig. 3a,b,c). However, *S. altissima* genotype A2 only showed an increase in P immobilization when decomposed with *S. gigantea* genotype G3 (Fig. 3b), and not G1 (Fig. 3d). These results show that focal genotype and neighbor genotype may interact to affect rates of P immobilization. This may be due to genotypic differences in resource use efficiency, or neighbor-induced changes to biomass allocation that influence a plant’s ability to tightly cycle P. However, this experiment was not designed to determine how neighbor genotype could influence P immobilization in mixed litterbags, and further experimentation will be required to elucidate these interactions. Recent research suggests that the effect size of inter- and intraspecific variation are similar with respect to ecosystem function [25], but the different drivers of N and P immobilization illustrate that the effect size of inter- and intraspecific variation may be dependent upon the ecosystem process in question and whether biotic interactions are considered. As the effects of plants on N and P cycling are trait mediated, it is likely that the amount of genotypic and species variation for traits related to nutrient cycling plays a large role in determining whether genotype or species identity affects the cycling of a particular nutrient.

### Indirect Genetic Effects Persist after Senescence and Affect Ecosystems

Indirect genetic effects are a fundamental element of the co-evolutionary process [35], through which the genotype of one individual influences the fitness and phenotype of associated interacting individuals [12]. Although Wolf’s (1998) definition of IGEs restricts the term to intraspecific interactions, the IIGEs we refer to here occur between members of different species. These interspecific indirect genetic effects (IIGEs) differ from standard IGEs because they have community level consequences through their effects on species interactions [13]. Both types of indirect genetic effects are fundamental to the coevolutionary process because, among other things, they alter the expected relationship between genotypes and phenotypes [12], and because they exist as both an environment and a selective force [36]. Therefore, IIGEs of a neighbor species that change the genotype frequencies of a focal species will alter the biotic environment, and the evolutionary conditions, experienced by both species. With respect to the current results, IIGEs occurred when the genotypic identity of neighbors influenced the phenotypes of focal plants by altering biomass production (shown in a previous study; [29]). These changes in plant traits altered litter quality; this led to “afterlife” effects on decomposition rate and N dynamics (Fig. 4). Although the r^2^ values for afterlife effects are relatively small, it is important to remember that that these values represent “extra” explanatory power beyond a model that only examined species and genotype identity on ecosystem processes. It also represents extra explanatory power beyond what is explained by abiotic factors such as temperature and moisture. Unlike abiotic environment effects, the afterlife effects of neighbor genotype have evolutionary implications and their importance should be interpreted in the context of how much extra information afterlife effects provide. In other words, the afterlife effects show that a given genotype affects ecosystem processes differently, depending on the traits it expressed while alive. Because the genetic component of these traits in conserved across replicates of the same genotype, it is environmental influences (which are partially due to IIGEs, as described earlier) that provide additional information about the ecosystem responses we measured.

We identified initial litter quality (lignin:N) as a potential mechanism for how changes in plant biomass traits could have afterlife effects on ecosystem processes. As plants produced more aboveground biomass and less coarse root biomass, litter quality increased (i.e., lower lignin:N; Table 2), and N immobilization also increased. Low coarse root biomass and high aboveground biomass were both also associated with increases in N immobilization. The greater overall N accumulation in high-quality litter could be due to its attractiveness to heterotrophic microbes, resulting in increased microbial biomass and N immobilization (e.g., [37]). Although we did not explicitly test how plant-neighbor interactions affected litter quality, increases in litter quality could be due to mechanisms that increase focal plant aboveground biomass, decrease focal plant coarse root biomass, or both. Competitive ability is thought to be related to a plant’s ability to reduce the concentration of limiting nutrients [38], [39] and because plants allocate resources to maximize the capture of limiting nutrients, neighbors that are weak competitors for N may allow focal plants to allocate less carbon to belowground structures. This could increase focal plant shoot to root ratio as more carbon is available for the production of aboveground biomass. In contrast with coarse root and aboveground biomass, we did not detect a correlation between rhizome biomass and litter quality, suggesting that the effects of rhizome biomass on decomposition rate are due to a different mechanism. One possibility is the translocation of nutrients from mature “mother” ramets to developing “daughter” ramets, which is common in clonal organisms (e.g., [40]), including *Solidago* [41]. If N translocation occurred, it could explain why plants with rhizome connections to many daughter plants have lower quality litter, and therefore slower decomposition rates, than plants with less rhizome biomass.

These results also provide insight into aboveground-belowground interactions by showing that “afterlife” effects can be initiated by IIGEs. Previous work had shown that species identity (e.g., [4]), interactions with herbivores [6]–[9], ozone [7] and UV radiation [10] could all initiate afterlife effects by changing litter quality. To our knowledge, our study is the first to show that IIGEs can also similarly initiate afterlife effects by changing litter quality, which represents an important advance as it suggests that ecosystem processes can be described as the gene-less products of direct (focal genotype) or indirect (neighbor genotype or IIGEs) genetic effects. Our previous work in *Solidago* showed that neighbor genotype identity can affect coarse root, rhizome, and aboveground biomass ([29], Fig. 4), all of which represent types of IIGEs. In this study we extended these results by showing that these IIGEs can also affect decomposition and nutrient dynamics by affecting plant litter chemistry. This holistic approach advances our understanding of aboveground-belowground interactions as it shows how plants’ living interactions influence the quality of their inputs to the organic matter pool, which can influence rates of litter decay, nutrient dynamics and localized nutrient cycles. Future work on ecosystem processes should be undertaken with the understanding that many biotic and abiotic environmental variables, including IIGEs, can drive trait expression at multiple stages of a plant’s life cycle, and these changes in trait expression can have important impacts on ecosystem processes.

## Materials and Methods

### Study Species and Experimental Design

*Solidago altissima* is a dominant species in abandoned agricultural fields where it can have large impacts on biodiversity and ecosystem function [22], [33]. Genotypes (i.e., intraspecific clonal families) display variation in biomass production, leaf size, green leaf N concentration, leaf litter decomposition and N release [21], [22], [42]. Although *S. altissima* and *S. gigantea* species are ecologically similar perennial plants, they differ in life-history traits [42], allocation of resources to different growth forms, and tolerance for variation in soil moisture [43].

In April 2008, a common garden experiment was established at Freels Bend on the reservation of Oak Ridge National Laboratory (Oak Ridge, TN) to examine the community and ecosystem level impacts of IIGEs in a *Solidago* sp. system. Freels Bend is public land and requires permission from Oak Ridge National Laboratory for access, but no permits are required. We did not sample protected species at the study site. This common garden included three locally collected genotypes (i.e., clonal lines) each of *S. altissima* and *S. gigantea*. The experimental treatments included genotype monocultures as well as all possible interspecific combinations of *S. altissima* and *S. gigantea* genotypes, planted together in 95 L pots. The genotypes were originally collected by G. M. Crutsinger from random locations around the study site at Freels Bend; sampled individuals from both species were carefully collected from unique connected genets that were at least 50–150 m apart [22]. The three *S. altissima* genotypes were originally collected and determined as unique genotypes using AFLP ([22] Supplementary Material); however, molecular data is unavailable for the *S. gigantea* genotypes. Although we used only three genotypes, this is sufficient for our purposes because we are not trying to characterize the variation present in populations of the two species, but rather trying to explore the emerging patterns that arise when genotypes interact in nature. All experimental plants were propagated from clonal lines of the genotypes described above. A 3-cm rhizome of each species and genotype was grown in greenhouses and watered as needed until the plants were ∼10 cm in height, at which point they were transplanted to the common garden experiment at Freels Bend. Plants were fertilized (24/8/16, Miracle-Gro, Marysville, OH, USA) once at the beginning of the experiment and watered as needed.

This setup included 6 monoculture treatments and 9 interspecific genotype combinations for a total of 15 treatments (n = 3 replicates per treatment). Four plants (either four of one genotype for monocultures, or two each of two genotypes for mixtures) were initially planted in each plot, but variation in density quickly occurred due to the clonal production of ramets. The plants were grown under competitive conditions, and were fertilized once (Miracle Gro, 24∶8:16 NPK ratio) at the beginning of the experiment. In October 2008, during leaf senescence, leaf litter was collected from these plants for a litter decomposition experiment (details below).

Litter mixing studies that explicitly examine intraspecific genetic variation face unique issues as genotypes are often morphologically indistinguishable to researchers, but may have chemical traits that make their individual and combined (i.e., genotype by genotype interactions) effects on decomposition unpredictable. The standard design for litter mixing studies involves the incubation of leaf litter in monoculture bags and mixture bags, followed by a comparison of the observed rates of decomposition and nutrient release with expected rates derived from mean monoculture results (e.g., [44], [45]). Generally, the species to be mixed are picked such that they can be visually identified and separated even late into the decomposition process (e.g., [46]). Under this standard design for mixture decomposition experiments, experiments that include phenotypically similar species (whose differences cannot be visually identified during the later stages of decomposition) would be unable to determine the mechanisms driving changes in decomposition and nutrient release rates. Therefore, the standard design would be unable to address at least one frequently proposed mechanism – the “priming” effect, through which high nutrient litter creates conditions that allow lower nutrient litter to decompose faster [9], [47].

We collected leaf litter by hand from senescing *S. altissima* and *S. gigantea* plants from the plant neighborhood experiment (described above) in October 2008, when the plants had been growing in the common garden for seven months. We pooled all plant litter from within each treatment; in other words, all litter collected from a given genotype-neighbor genotype pair was pooled and mixed before chemical analyses, and before weighing out litter to be used in the experiment. We took this approach because the majority of plants had not produced enough litter to be used as individual units in the decomposition experiment. For this reason, with respect to afterlife effects, we do not examine the relationship between plant traits and ecosystem processes at the level of individual plants, but rather we make the comparison using genotype-neighbor genotype mean values (i.e., the mean trait value of *S. altissima* genotype #1 when grown with *S. gigantea* genotype #2, or in the case of monocultures the mean trait value of *S. gigantea* genotype #3 when grown with *S. gigantea* genotype #3).

We used a “bag within a bag” design that allowed us to segregate litter by type (*sensu* [48]). This design included smaller bags and larger bags. Smaller bags were used to partition leaf litter by species and genotype identity, and larger bags enclosed two smaller bags to form each experimental replicate. We controlled for position of smaller bags (i.e., top vs. bottom) for equal representation. The larger, exterior bags were 5 cm×5 cm and were constructed using large diameter mesh (2 mm) on the top to allow access to decomposer organisms, and small diameter mesh (0.25 mm) on the bottom to prevent loss of litter from the bag by fragmentation. The smaller, interior bags (3 cm×3 cm) were made using large meshed material on both sides (2 mm). This was done to maximize litter interactions between the smaller, interior bags while allowing us to keep the material separate throughout decomposition. The interior bags were filled with 1.5 g of leaf litter according to the specific experimental treatments, identified with a labeling tag and placed inside the exterior bags. We recognize, and emphasize, that this is a conservative test for species interactions, genotype interactions, and non-additivity because the litter types are not as thoroughly mixed as in natural systems, and results should be interpreted in consideration of this fact. The design included six monoculture treatments in which the focal and neighbor genotypes had the same genotype identity, and 5 genotype mixture treatments in which the focal and neighbor genotypes had different genotype identities. We only used 5 of the possible 9 interspecific genotype combinations because we could not obtain sufficient amounts of leaf litter from the other 4 combinations. Unfortunately, this limitation makes it impossible to test for the effects of “neighbor genotype” on ecosystem processes, as different neighbor genotypes are present for each focal genotype. Therefore, no “neighbor genotype” tests are presented here. However, we detected significant IIGEs in a previous experiment [29] and here we discuss how these IIGEs affected ecosystem processes after plant senescence. Each of the 11 treatments was replicated three times over three collection dates for a total of 99 large and 198 small litter bags. The litterbags were placed in the field to decompose on 19 December 2008, and one third of the bags were collected on each of the following dates: 10 February 2009, 25 April 2009, and 22 August 2009, after two, four and eight months in the field, respectively, and after eight months the litter had lost up to 80% of the original mass. We blocked the experimental design by placing the bags at three locations approximately five meters from each other (ten meters maximum for the two blocks that were furthest away from each other) at the same site at Freels Bend where the plants were grown. However, we found that including the blocking factor in our model did not affect our results or conclusions.

After each collection date, the litterbags were removed from the field and all soil and biotic contaminants were removed by hand. The samples were then air-dried in paper sacks, individually weighed and then ground through a 40 mesh screen with a Wiley Mill. Subsamples of the ground leaf material were separately ashed (500°C for 1 h) and oven-dried (70°C for 48 h). All final weights are expressed on an ash-free, oven-dry mass basis (AFODM). Nutrient dynamics (i.e., nutrient immobilization or loss) were assessed for each sample by examining total N and phosphorus (P) concentrations in leaves from each genotype and species individually (i.e., from each of the individual bag samples), initially (time 0) and after each collection date. The remainder of the ground initial litter material was stored at 4°C until lignin analyses could be conducted.

Litter chemical parameters at time 0 were quantified to determine if differences among genotypes influenced litter lignin, N and P content. Initial litter lignin was determined using the acid-fiber detergent method using an Ankom 200 fiber analyzer (Ankom Technology, Macedon NY); *Quercus rubrum* leaf litter was used as a standard. Total litter N and P were determined on the initial samples as well as each collection date by modified micro-Kjeldahl digestion [49] and analyzed on a Lachat AE Flow Injection Analyzer (Lachat Instruments, Inc., Loveland, CO, USA) using the salicylate and molybdate-ascorbic acid methods, respectively; apple leaves (*Malus* sp. mixture) were used as a standard (SRM 1515, NIST, Gaithersburg, MD, USA). Our estimates of litter nutrient concentration over time include N and P present in both plant material and microbial decomposers.

### Statistical Methods

To determine genotype and species-level effects on initial litter chemistry, we used ANOVAs with species identity, and genotype nested within species, as fixed factors. We did not incorporate the possible effects of genotype by neighbor genotype interactions on plant chemistry in this analysis, because of our limited number of genotype-neighbor genotype pairs. We also used an ANOVA approach to analyze patterns of mass loss and nutrient concentration over time (*sensu* [50]) with the factors time, species, and neighbor species in a full factorial design. We used these factors to assess the contribution of species interactions to ecosystem processes, and allowed all factors to interact to see if the influence of species interactions changed over the course of decomposition. For mass loss, we did not detect any interaction terms including time, suggesting that a single exponential approach was sufficient to model decomposition. To calculate decomposition rate constants (k), we determined the linear slope of the natural-log-transformed mass loss data. The relationship between time and percent mass remaining was significant for every species/neighbor species pair, with r2 values ranging from 0.686 to 0.828. To determine the effects of genotype identity on decomposition and nutrient dynamics, we repeated this analysis with the factors time, species, and genotype nested within species. All analyses were conducted in JMP 9.0 (SAS Institute 2010).

To examine the non-additive effects of genotype mixtures on mass loss total N and P immobilization, we compared our observed values to additive expectations. The relative contribution of each genotype to nutrient dynamics changed over time, as *S. altissima* lost mass faster than *S. gigantea*, but our expectations were calculated based on initial conditions when *S. altissima* and *S. gigantea* were present in equal amounts. We calculated expected values for each mixture as the average of the component genotypes in monoculture [45]. For decomposition rate, we compared observed and expected k-constants. For nutrient dynamics, we used nutrient concentrations, averaged over time and relative to initial values [18]. If our expected values fell within the 95% confidence intervals of our observed values, we called the effect “additive,” and otherwise we called the effect “non-additive.” We stress that if two IIGEs of opposite signs are present, then they may counteract each other’s effects and leave the plot-level measurements within the range of expected values. The result of this can be that non-additivity is not detected at the “large bag” level despite the occurrence of IIGEs between the two genotypes within the bag.

To determine “afterlife” effects, we examined previously published above- and belowground biomass data measured throughout the 2009 growing season. The previously published biomass data was collected from plants growing in the same common garden from which senescing leaves were collected. From this previous work, we knew that neighbor genotype identity significantly affected three traits – rhizome biomass, coarse root biomass, and aboveground vegetative biomass [29], so we calculated means for genotype-neighbor genotype pairs (e.g., all measurements from genotype A1 grown with genotype G1) for these traits and compared them to genotype-neighbor genotype pair means for decomposition rate, average N change (%) and average P change (%) (across all collection dates, relative to initial values for both nutrients). We took this approach because, within a genotype, initial litter N and P were variable depending on the neighbor genotype with which it was grown. In other words, we used genotype-neighbor genotype means to obtain an accurate starting point from which to assess change in N and P for the purposes of determining afterlife effects. We used mean values for focal genotype-neighbor genotype pairs because litter for decomposition had been pooled, and we could not pair decomposition data points with a matching “growing season” data point. We transformed the focal genotype-neighbor genotype means to meet assumptions of normality, and then used generalized linear models (GLMs) with a normal distribution and identity link function. Our model included the following factors: rhizome biomass, coarse root biomass, aboveground vegetative biomass, species identity, and genotype nested within species. We used these factors to separately predict litter quality (lignin:N) decomposition rate constant (k), average N change (%), and average P change (%). Again, factors were chosen based on plant traits we knew to be affected by IIGEs, and we included species and neighbor genotype to ensure that plant biomass traits were responsible for changing decomposition rate and nutrient dynamics even after correcting for genotype and species-level differences. Finally, we included litter quality because IIGEs affecting litter quality would provide a mechanistic link between plant biomass traits and ecosystem processes.

## References

[1] Van der Putten WH, Vet LEM, Harvey JA, Wackers FL (2001) Linking above- and belowground multitrophic interactions of plants, herbivores, pathogens, and their antagonists. Trends Ecol Evol 16: 547–554.

[2] Wardle DA, Bardgett RD, Klironomos JN, Setala H, van der Putten WH, et al (2004) Ecological linkages between aboveground and belowground biota. Science 304: 1629–1633. 15192218 10.1126/science.1094875

[3] Bardgett RD, Wardle DA (2010) Aboveground-belowground linkages: biotic interactions, ecosystem processes, and global change. Oxford: Oxford University Press. 320 p.

[4] Melillo JM, Aber JD, Muratore JF (1982) Nitrogen and lignin control of hardwood leaf litter decomposition dynamics. Ecology 63: 621–626.

[5] Whitham TG, Gehring CA, Lamit LJ, Wojtowicz T, Evans LM, et al (2012) Community specificity: life and afterlife effects of genes. Trends Plant Sci 16: 271–281. 10.1016/j.tplants.2012.01.005 22322002

[6] Choudhury D (1988) Herbivore induced changes in leaf-litter resource quality – a neglected aspect of herbivory in ecosystem nutrient dynamics. Oikos 51: 389–393.

[7] Findlay S, Carreiro M, Krischik V, Jones CG (1996) Effects of damage to living plants on leaf litter quality. Ecol Appl 6: 269–275.

[8] Wardle DA, Bonner KI, Barker GM (2002) Linkages between plant litter decomposition, litter quality, and vegetation responses to herbivores. Func Ecol 16: 585–595.

[9] Schweitzer JA, Bailey JK, Hart SC, Whitham TG (2005) Non-additive effects of mixing cottonwood genotypes on litter decomposition and nutrient dynamics. Ecology 86: 2834–2840.

[10] Caldwell M, Teramura AH, Tevini M, Bornman JF, Bjorn LO, et al (1995) Effects of increased solar ultraviolet radiation on terrestrial plants. Ambio 24: 166–173.

[11] Hobbie SE (1992) Effects of plant-species on nutrient cycling. Trends Ecol Evol 7: 336–339. 21236058 10.1016/0169-5347(92)90126-V

[12] Wolf JB, Brodie ED, Cheverud JM, Moore AJ, Wade MJ (1998) The evolutionary consequences of indirect genetic effects. Trends Ecol Evol 13: 64–59. 21238202 10.1016/s0169-5347(97)01233-0

[13] Shuster SM, Lonsdorf EV, Wimp GM, Bailey JK, Whitham TG (2006) Community heritability measures the evolutionary consequences of indirect genetic effects on community structure. Evolution 60: 991–1003. 16817539

[14] Moya-Larano J (2012) O matrices and eco-evolutionary dynamics. Trends Ecol Evol 27: 139–140. 22196740 10.1016/j.tree.2011.11.013

[15] Gartner TB, Cardon ZG (2004) Decomposition dynamics in mixed-species leaf litter. Oikos 104: 230–246.

[16] Hättenschwiler S, Tiunov AV, Scheu S (2005) Biodiversity and litter decomposition in terrestrial ecosystems. Annu Rev Ecol Evol S 36: 191–218.

[17] Gessner MO, Swan CM, Dang CK, McKie BG, Bardgett RD, et al (2010) Diversity meets decomposition. Trends Ecol Evol 25: 372–380. 20189677 10.1016/j.tree.2010.01.010

[18] Madritch M, Donaldson JR, Lindroth RL (2006) Genetic identity of Populus tremuloides litter influences decomposition and nutrient release in a mixed forest stand. Ecosystems 9: 528–537.

[19] Hansen RA, Coleman DC (1998) Litter complexity and composition are determinants of the diversity and species composition of oribatid mites (Acari : Oribatida) in litterbags. Appl Soil Ecol 9: 17–23.

[20] Whitham TG, Young WP, Martinsen GD, Gehring CA, Schweitzer JA, et al (2003) Community and ecosystem genetics: A consequence of the extended phenotype. Ecology 84: 559–573.

[21] Schweitzer JA, Bailey JK, Hart SC, Whitham TG (2005) Nonadditive effects of mixing cottonwood genotypes on litter decomposition and nutrient dynamics. Ecology 86: 2834–2840.

[22] Crutsinger GM, Collins MD, Fordyce JA, Gompert Z, Nice CC, et al (2006) Plant genotypic diversity predicts community structure and governs an ecosystem process. Science 313: 966–968. 16917062 10.1126/science.1128326

[23] Whitham TG, Bailey JK. Schweitzer JA, Shuster SM, Bangert RK, et al (2006) A framework for community and ecosystem genetics: from genes to ecosystems. Nat Rev Genet 7: 510–523. 16778835 10.1038/nrg1877

[24] Fridley JD, Grime JP, Bilton M (2007) Genotypic identity of interspecific neighbours mediates plant responses to competition and environmental variation in a species-rich grassland. J Ecol 95: 908–915.

[25] Bailey JK, Schweitzer JA, Ubeda F, Koricheva J, LeRoy CJ, et al (2009) From genes to ecosystems: A synthesis of the effects of plant genetic factors across levels of organization. Philos T Roy Soc B 364: 1607–1616. 10.1098/rstb.2008.0336 PMC2690499 19414474

[26] Bossdorf O, Shuja Z, Banta JA (2009) Genotype and maternal environment affect belowground interactions between Arabidopsis thaliana and its competitors. Oikos 118: 1541–1551.

[27] Genung MA, Lessard JP, Brown CB, Bunn WA, Cregger MA, et al (2010) Non-additive effects of genotypic diversity increase floral abundance and abundance of floral visitors. PLoS ONE 5: e8711. 20090850 10.1371/journal.pone.0008711 PMC2806830

[28] Madritch MD, Lindroth RL (2011) Soil microbial communities adapt to genetic variation in leaf litter inputs. Oikos 120: 1696–1704.

[29] Genung MA, Bailey JK, Schweitzer JA (2012) Welcome to the neighbourhood: interspecific genotype by genotype interactions in Solidago influence above- and belowground biomass and associated communities. Ecol Lett 15: 65–73. 22070740 10.1111/j.1461-0248.2011.01710.x

[30] Turkington R, Harper JL (1979) Growth, distribution and neighbor relationships of Trifolium repens in a permanent pasture. 4. Fine-scale biotic differentiation. J Ecol 67: 245–254.

[31] Aarssen LW, Turkington R (1985) Biotic specialization between neighboring genotypes in Lolium perenne and Trifolium repens from a permanent pasture. J Ecol 73: 605–614.

[32] Wade MJ (2007) The co-evolutionary genetics of ecological communities. Nat Rev Genet 8: 185–195. 17279094 10.1038/nrg2031

[33] Maddox GD, Root RB (1987) Resistance to 16 diverse species of herbivorous insects within a population of goldenrod, Solidago altissima: genetic variation and heritability. Oecologia 72: 8–14. 28312889 10.1007/BF00385037

[34] Finzi AC, Canham CD (1998) Non-additive effects of litter mixtures on net N mineralization in a southern New England forest. Forest Ecol Manag 105: 129–136.

[35] Thompson JN (2005) The geographic mosaic of coevolution. Chicago: University of Chicago Press. 400 p.

[36] Dawkins R (1982) The extended phenotype: the gene as the unit of selection. Oxford: Oxford University Press. 307 p.

[37] Blair JM, Crossley DA, Callaham LC (1992) Effects of litter quality and microarthropods on N dynamics and retention of exogenous N-15 in decomposing litter. Biol Fert Soils 12: 241–252.

[38] O’Brien WJ (1974) Dynamics of nutrient limitation of phytoplankton algae – model reconsidered. Ecology 55: 135–141.

[39] Tilman D, Kilham SS, Kilham P (1982) Phytoplankton community ecology – the role of limiting nutrients. Annu Rev Ecol Syst 13: 349–372.

[40] Caraco T, Kelly CK (1991) On the adaptive value of physiological integration in clonal plants. Ecology 72: 81–93.

[41] Abrahamson WG, Anderson SS, McKrea KD (1991) Clonal integration – nutrient sharing between sister ramets of Solidago altissima (Compositae). Am J Bot 78: 1508–1514.

[42] Abrahamson WG, Weis AE (1997) Monographs in Population Biology, Volume 29: Evolutionary ecology across three trophic levels: goldenrods, gallmakers, and natural enemies. Princeton: Princeton University Press. 456 p.

[43] Abrahamson WG, Dobley KB, Houseknecht HR, Pecone CA (2005) Ecological divergence among five co-occuring species of old-field goldenrods. Plant Ecol 177: 43–56.

[44] Blair JM, Parmelee RW, Beare MH (1990) Decay rates, nitrogen fluxes, and decomposer communities of single- and mixed-species foliar litter. Ecology 71: 1976–1985.

[45] Wardle DA, Bonner KI, Nicholson KS (1997) Biodiversity and plant litter: Experimental evidence which does not support the view that enhanced species richness improves ecosystem function. Oikos 79: 247–258.

[46] Chapman SK, Newman GS (2010) Biodiversity at the plant-soil interface: microbial abundance and community structure respond to litter mixing. Oecologia 162: 763–769. 19921274 10.1007/s00442-009-1498-3

[47] Briones MJI, Ineson P (1996) Decomposition of eucalyptus leaves in litter mixtures. Soil Biol Biochem 28: 1381–1388.

[48] Wardle DA, Nilsson MC, Zackrisson O, Gallet C (2003) Determinants of litter mixing effects in a Swedish boreal forest. Soil Biol Biochem 35: 827–835.

[49] Parkinson JA, Allen SE (1975) Wet oxidation procedure suitable for determination of nitrogen and mineral nutrients in biological-material. Commun Soil Sci Plan 6: 1–11.

[50] Wieder RK, Lang GE (1982) A critique of the analytical methods used in examining decomposition data obtained from litter bags. Ecology 63: 1636–1642.

